# Is alexithymia associated with weight-related self-esteem after metabolic and bariatric surgery: A cross-sectional study

**DOI:** 10.1097/MD.0000000000047191

**Published:** 2026-01-16

**Authors:** Merve Şahin Can, Ferhat Çay

**Affiliations:** a Faculty of Medicine, Department of Psychiatry, Balikesir University, Balikesir, Turkey; b Faculty of Medicine, Department of General Surgery, Balikesir University, Balikesir, Turkey.

**Keywords:** alexithymia, esteem, metabolic bariatric surgery, related self, weight

## Abstract

Metabolic and bariatric surgery has significant positive effects on quality of life. The aim of the present study is to assess whether or not alexithymia is associated with weight-related self-esteem levels which is a concept assessed in quality of life after metabolic bariatric surgery. A cross-sectional study conducted at a university hospital. Before the surgery, patients who had no active psychiatric diagnosis or were not on psychiatric medication filled out the Hamilton anxiety rating scale and Hamilton depression rating scales. Total of 119 patients were revisited between the sixth and twelfth months after surgery and filled out a socio-demographic data form, the Hamilton anxiety rating scale, the Hamilton depression rating scale, the impact of weight on quality-of-life scale – self-esteem subscale (IWQOL-Lite), and the Toronto alexithymia scale. The mean time elapsed after the postoperative evaluation was 8.7 ± 3.1 months. About 29.5% of the patients applying to bariatric surgery were alexithymic. The mean scores on the HAM-D, HAM-A, difficulty identifying feelings, difficulty describing feelings, and externally oriented thinking scales of the patients were significantly higher in alexithymic patients (*P* = .008; *P* = .004; *P* < .001; *P* < .001; *P* = .004, respectively). Additionally, the IWQOL-Lite scores of alexithymic individuals were statistically significantly lower than those of the group without alexithymia (*P* = .004). A weak negative correlation was found between the score of IWQOL-Lite self-esteem and the total Toronto alexithymia scale-20 item score and difficulty describing feelings value (*r* = −0.185, *P* = .044; *r* = −0.209, *P* = .023, respectively). The present study supported that individuals with high alexithymic characteristics have lower weight-related self-esteem after the surgery, and further studies are needed to elucidate the causal relationship of this condition.

## 1. Introduction

The prevalence of obesity, a major global public health concern, has been steadily increasing. Many studies have reported the negative impact of obesity on quality of life by reducing physical, social, and occupational functioning, along with other disorders (cardiovascular, endocrine, metabolic, etc).^[1-4]^ Surgical interventions are used as an effective method to achieve weight loss in patients who are unresponsive to traditional treatment. Metabolic and bariatric surgery has significant effects on psychosocial health and quality of life, as well as being effective in ameliorating obesity-induced cardiac and metabolic pathologies. Several studies examining the quality of life and related factors after bariatric surgery using different scales have frequently assessed age, gender, preoperative body mass index, comorbidities, weight loss with surgical intervention, selected surgical method, and complications, and different conclusions have been drawn.^[5-14]^ Weight-related self-esteem refers to the effect of body shape and weight on self-concept and self-esteem and is considered as a subset of quality of life. In particular, there are studies indicating that assessing weight-related self-esteem is more significant than assessing global self-esteem in individuals suffering from obesity and/or eating disorders with weight-related concerns.^[15,16]^ Felske et al stated that weight-related self-esteem also improved after bariatric surgery and correlated this circumstance with reduced concern about body and shape, independent of weight loss.^[17]^ However, another study reported that although self-esteem improved in the first year following bariatric surgery, it was still lower than in the control group.^[18]^ Burgmer et al reported that the maximum positive change in self-esteem after surgery was noted in the first year, and as weight loss increased, there was an improvement in self-esteem.^[19]^ Therefore, it is still unclear how bariatric surgery affects weight-related self-esteem, and further research is necessary to understand the psychological factors that could affect this condition. The concept of alexithymia, defined as difficulty in identifying and describing emotions, is more frequently observed in individuals with obesity compared to the general population. It has been shown to negatively affect the mid-term outcomes of metabolic and bariatric surgery and moreover its relationship with quality of life is not sufficiently taken into account in the clinical practice.^[20,21]^ Therefore, in this study we aimed to investigate whether alexithymia is associated with weight-related self-esteem levels in individuals after metabolic and bariatric surgery. Our hypothesis was that the weight-related self-esteem level would be lower in individuals with high alexithymic traits compared to individuals without alexithymia.

## 2. Materials and methods

This cross-sectional study was conducted in the psychiatric outpatient clinics of the Health Application and Research Hospital at Balikesir University, and 125 bariatric surgery candidates aged between 18 and 65 years who met the inclusion criteria were included. The inclusion criteria were determined as follows; voluntarily agreeing to participate in the study, being literate, and a first application for bariatric surgery. Patients with an active psychiatric diagnosis or those on psychiatric medication were excluded from the study. The patients’ preoperative and postoperative depression and anxiety levels were assessed using the Hamilton depression rating scale (HAM-D) and Hamilton anxiety rating scale (HAM-A) and 2 patients with clinically significant scores in the preoperative period and 4 patients with clinically significant scores in the postoperative period were excluded from the study. A total of 119 patients were revisited between 6 and 12 months after surgery and completed the sociodemographic data form, the impact of weight on quality-of-life scale – self-esteem subscale (IWQOL-Lite), and the Toronto alexithymia scale (TAS-20) as self-reported. The study was approved by the Balikesir University Local Ethics Committee (No. 2022-56), and all participants provided written informed consent.

### 2.1. Socio-demographic data form

The researchers prepared this form, which included questions about age, height, weight, marital status, history of chronic disease, history of psychiatric disease, medications used, and alcohol and cigarette use.

### 2.2. Hamilton depression rating scale (HAM-D)

It was published by Max Hamilton in 1960.^[22]^ It consists of 17 items questioning symptoms of depression in the last week. The highest score is 53. About 0 to 7 points indicate no depression, 8 to 13 points indicate mild depression, 14 to 18 points indicate moderate depression, 19 to 22 points indicate severe depression, and 23 and above indicate very severe depression. The Turkish validity and reliability study of the scale was conducted by Akdemir et al.^[23]^

### 2.3. Hamilton anxiety rating scale (HAM-A)

It is a scale developed by Hamilton in 1959 to determine the severity of anxiety.^[24]^ It consists of 14 items assessing physical and psychic symptoms of anxiety. Items are rated between 0 and 4 points according to symptom severity. About 0 to 5 points are considered normal anxiety, 6 to 14 points are considered mild anxiety, 15 and above are considered severe anxiety. It is Turkish reliability and validity study was conducted by Yazici et al.^[25]^

### 2.4. Impact of weight on quality-of-life scale – Lite form (IWQOL-Lite)

Kolotkin et al developed the IWQOL-Lite by revising the longer version of IWQOL in 200.^[26]^ The IWQOL-Lite scale consists of 31 items and 5 subscales, including physical functions (11 items), self-esteem (7 items), sexual life (4 items), public distress (5 items), and work (4 items). The total and subscale scores of the IWQOL-Lite scale are calculated using a formula developed specifically for the scale. According to the scoring of the scale, lower scores signify that the quality-of-life falls; whereas, higher scores indicate that the quality of life rises. In 2011, Çömlekçi and Özcan conducted the validity and reliability study of the scale in Turkiye and adapted it as Turkish IWQOL-Lite, which consists of 29 items.^[27]^

### 2.5. Toronto alexithymia scale (TAS-20)

TAS-20 was developed by Bagby et al^[28]^. The Turkish adaptation, validity and reliability study of the scale was conducted by Güleç et al.^[29]^ Subscales of the scale are difficulty identifying feelings (DIF), difficulty describing feelings (DDF), externally-oriented thinking (EOT). High scores indicate a high level of alexithymia. According to the authors, it would be appropriate to take “51” as the lower value to avoid missing alexithymia.

### 2.6. Statistical analysis

For statistical analysis of the data, the SPSS (Statistical Package for the Social Sciences) 25.0 packaged software (IBM Corp., Armonk) was used. While categorical data were summarized as numbers and percentages, continuous data were summarized as means and standard deviations (median and minimum-maximum when necessary). The chi-square test was used to compare categorical data. The Kolmogorov–Smirnov test was run to determine whether or not the parameters were normally distributed. The Mann–Whitney *U* test was used for the parameters that were not normally distributed. The Spearman’s rho correlation test was run to determine the correlation between continuous measurement parameters. The statistical significance level was accepted as 0.05 for all tests. A priori power analysis conducted using G*Power, assuming a medium effect size with α = 0.05 and 80% power, indicated a required minimum of 110 participants.

## 3. Results

A total of 119 individuals who underwent bariatric surgery were assessed in the study. The mean age of the participants was 37.2 ± 11.6 years, and 90 (75.6%) of them were female. The preoperative mean body mass index value of the patients was 45.9 ± 7.1. Preoperative HAM-D and HAM-A mean scores of the participants were 2.31 ± 2.9 and 3.26 ± 4.8 respectively.

The mean time elapsed after the postoperative evaluation of the individuals was 8.7 ± 3.1 months. When asked subjectively about their overall satisfaction level, 105 (88.2%) of the patients stated that they were satisfied with their weight after surgery. The postoperative IWQOL-Lite-self-esteem subscale mean score was 93.39 ± 9.1.

The participants were divided into 2 groups; alexithymic (N = 35) and non-alexithymic (N = 84) according to the threshold value of 51 points in TAS-20, the rate of alexithymic individuals among the people who applied to bariatric surgery was 29.5%. While 77% of alexithymic individuals (N = 27) stated that they were satisfied with their weight after surgery, 92.9% of non-alexithymic individuals (N = 78) stated that they were subjectively satisfied with their weight after surgery (*P* = .015). No significant difference was detected in the 2 groups with respect to gender (*P* = .236). The mean scores of the HAM-D, HAM-A, DIF, DDF, and EOT of the patients were significantly higher in alexithymic patients (*P* = .008; *P* = .004; *P* < .001; *P* < .001; *P* = .004, respectively). Additionally, the weight-related self-esteem scores of alexithymic individuals were statistically significantly lower than those of the group without alexithymia (*P* = .004; Table 1).

**Table 1:** The clinical characteristics of the groups and their postoperative mean scores on the clinical evaluation scale.

	TAS score < 50 (n = 84)	TAS score ≥ 51 (n = 35)	*P*
Gender			
Female	61 (72.6)	29 (82.9)	.236
Male	23 (27.4)	6 (17.1)	
Age	35.9 ± 10.8	40.5 ± 12.7	**.049** *
Height	164.6 ± 9.7	162.9 ± 10.8	.262
Weight	122.8 ± 20.9	126.2 ± 28.3	.811
HAM-D	1.86 ± 2.5	3.37 ± 3.7	**.008** *
HAM-A	2.61 ± 4.2	4.80 ± 5.8	**.004** *
DIF	10.5 ± 3.2	18.8 ± 5.9	**<.001** **
DDF	8.69 ± 2.4	14.4 ± 3.7	**<.001** **
EOT	20.8 ± 4.4	23.4 ± 3.8	**.004** *
Weight related self-esteem	95.15 ± 7.1	89.17 ± 11.55	**.004** *
Time elapsed after the surgery	8.80 ± 3.1	8.58 ± 3.1	.780

BMI = body mass index, DDF = difficulty describing feelings, DIF = difficulty identifying feelings, EOT = externally-oriented thinking, HAM-A = Hamilton anxiety rating scale, HAM-D = Hamilton depression rating scale, TAS = Toronto alexythymia scale.

Mean ± SD = Mann–Whitney *U*, + = chi-square. Bold values indicates statistically significant results (*P* < .05).

* *P* < .05.

** *P* < .001.

Finally, when analyzing the correlation between IWQOL-Lite self-esteem and TAS-20 scores, a weak negative (linear) correlation (*r* = −0.185, *P* = .044; *r* = −0.209, *P* = .023, respectively) was found between IWQOL-Lite self-esteem and the total TAS-20 score and value of its subscale DDF, and no significant correlation was found between self-esteem and DIF and EOT values (*r* = −0.159, *r* = 0.052, respectively; Table 2).

**Table 2:** The correlation between the TAS-20 and IWQOL-Lite self-esteem scores of the participants.

Alexithymia	Weight related self-esteem	
	*r*	*P*
Difficulty identifying feelings	−0.159	.083
Difficulty describing feelings	−0.209*	**.023**
Externally-oriented thinking	0.052	.574
Total alexithymia	−0.185*	**.044**

IWQOL-Lite = impact of weight on quality of life - lite form, TAS-20 = Toronto alexithymia scale-20 item.

*r* = spearman’s rho correlation, + = chi-square. Bold values indicates statistically significant results (*P* < .05).

* *P* < .05.

## 4. Discussion

The present study examined the correlation between alexithymic traits and weight-related self-esteem after the surgery, and the findings were discussed in the light of the literature. Main findings of the present study can be briefly summarized as follows: Approximately 30% of the people who underwent bariatric surgery had alexithymic traits. Individuals in this group had lower levels of weight-related self-esteem after the surgery, and weight-related self-esteem was negatively correlated with the difficulty describing feelings, a subscale of alexithymia.

The global rise in the prevalence of obesity has increased the demands for metabolic and bariatric surgery. The positive contributions of surgery to both weight loss and the prognosis of obesity-related chronic diseases undoubtedly provide individuals with better physical functions and quality of life.^[12,30]^ In the literature, various scales assessing the overall quality of life have been used in studies examining the quality of life after bariatric surgery. For example, a study using the Moorehead Ardelt Quality of Life Questionnaire II reported that the improvement in quality of life after surgery was nonlinear, that changes were different between men and women, and the fifteenth to eighteenth months were the critical periods.^[10]^ However, the use of overall quality-of-life scales in groups with weight-related concerns has been gradually superseded by studies evaluating weight-related quality of life.^[15]^ Karayurt et al found that total mean score of IWQOL was 75.12 ± 20.14 after surgery and all subscales were moderate.^[31]^ Another study conducted by Sarwer et al, reported that bariatric surgery significantly improved all specific domains of weight-related quality of life.^[32]^ Weight-related self-esteem is a psychosocial domain evaluated under the subheading of weight-related quality of life. Preoperative weight-related self-esteem and depression symptoms have been identified as potential predictors of persistent postoperative shape and weight concerns, suggesting that self-esteem may be a crucial target that can improve outcomes throughout the surgical process.^[17]^ Moreover, low preoperative self-esteem was found to be negatively correlated with postoperative weight regain.^[33]^ Eroğlu et al reported low results in their study examining quality of life and self-esteem in candidates for surgery.^[34]^ Studies on self-esteem in the postoperative period have been contradictory. One study showed no favorable change in self-esteem at 6 months after surgery compared to the previous period.^[35]^ Another study showed an improvement in self-esteem at the end of a 1-year follow-up, but this improvement was not statistically significant. In these studies a generic scale was used rather than a weight-specific scale to assess self-esteem, which may have affected results.^[36]^ Most probably, psychosocial needs that are not met after surgery – weight regain, negative changes related to the body, or the consequences of surgery that fail to satisfy expectations – may impair self-esteem. However, the lack of studies in which psychological factors that contribute to this issue are investigated draws attention. The concept of alexithymia is frequently studied in psychiatric diseases and obesity. Different results have been documented in studies on the rates of obesity accompanied by alexithymia. Marechal et al found that this rate was 42.9% in obese individuals applying to surgical centers.^[37]^ Research has shown that alexithymia is a common psychological feature that may affect self-esteem and eating behavior.^[38]^ Li et al determined a significant correlation between alexithymia and food addiction and indicated that it could be evaluated in clinical procedures.^[39]^ A study conducted on candidates for bariatric surgery reported that alexithymia levels were higher in food addicts, and alexithymia was defined as a partial mediator factor between adult attention deficit and food addiction among them.^[40]^ The overall TAS-20 scale and its subscales “Difficulty identifying feelings,” and “Externally-oriented thinking” were also found to be negatively correlated with adherence to the postoperative nutrition plan in candidates for bariatric surgery.^[41]^ A long-term follow-up study reported that alexithymia was negatively correlated with weight loss between 24 and 30 months after surgery, but this correlation was not observed in the early period.^[21]^ Therefore, the effect of alexithymia on the outcomes of bariatric surgery is considered to be a factor that should be investigated. The present study revealed that alexithymic individuals had lower levels of weight-related self-esteem. This may be caused by the subjective postoperative satisfaction levels of alexithymic individuals who have difficulty describing their feelings. In the literature, it was hypothesized that alexithymic difficulty in the cognitive processing of emotions led to an intensification of bodily sensations associated with emotional arousal.^[42]^ Furthermore, another study reported that the level of alexithymia was strongly correlated with a high level of self-perception as “obese.”^[43]^ This suggests that alexithymia might be one of the reasons for lower self-esteem. However, further studies are needed to elucidate the causal relationship between alexithymia and weight-related self-esteem.

The present study has some limitations. Firstly, the weight-related self-esteem levels of the patients were not assessed preoperatively; therefore, it is not possible to determine whether these levels improved after metabolic and bariatric surgery or whether they changed specifically in alexithymic individuals. Thus, future studies incorporating preoperative assessments of quality of life and alexithymia are needed to provide a more comprehensive understanding of the trajectory of change and the role of psychological factors throughout the surgical process. Secondly, we assessed only the self-esteem subscale of quality of life. Therefore, large-sample studies including other subscales are warranted to better clarify potential associations. Finally, in our clinic, HAM-D and HAM-A are routinely used as standardized clinician-administered scales; however, considering obesity as a medical illness, future studies employing HADS may also be appropriate in bariatric populations.

## 5. Conclusion

However, as the first study to evaluate the correlation between alexithymia and weight related-self-esteem after surgery, the present study is considered to contribute to the investigation of psychosocial factors that may affect surgical outcomes.

## Author contributions

**Conceptualization:** Merve Şahin Can, Ferhat Çay.

**Data curation:** Merve Şahin Can, Ferhat Çay.

**Formal analysis:** Merve Şahin Can.

**Investigation:** Merve Şahin Can.

**Methodology:** Merve Şahin Can.

**Resources:** Merve Şahin Can.

**Supervision:** Ferhat Çay.

**Writing – original draft:** Merve Şahin Can, Ferhat Çay.

**Writing – review & editing:** Merve Şahin Can.
